# Crystal structures of two copper(I)–6,6′-dimethyl-2,2′-bipyridyl (dmbpy) compounds, [Cu(dmbpy)_2_]_2_[*M*F_6_]·*x*H_2_O (*M* = Zr, Hf; *x* = 1.134, 0.671)

**DOI:** 10.1107/S2056989021007295

**Published:** 2021-07-20

**Authors:** Yiran Wang, Matthew L. Nisbet, Kenneth R. Poeppelmeier

**Affiliations:** a Northwestern University, 2145 Sheridan Road, Evanston, IL 60208, USA

**Keywords:** crystal structures, Cu^I^ complex, *d* ^0^ early transition metals, hydro­thermal synthesis

## Abstract

The syntheses and crystal structures of two bimetallic mol­ecular compounds, namely, bis[bis­(6,6′-dimethyl-2,2′-bi­pyridine)­copper(I)] hexa­fluorido­zir­con­ate(IV) 1.134-hydrate, [Cu(dmbpy)_2_]_2_[ZrF_6_]·1.134H_2_O (dmbpy = 6,6′-di­methyl-2,2′-bipyri­dyl, C_12_H_12_N_2_), (I), and bis[bis­(6,6′-dimethyl-2,2′-bi­pyr­idine)­copper(I)] hexa­fluorido­hafnate(IV) 0.671-hydrate, [Cu(dmbpy)_2_]_2_[HfF_6_]·0.671H_2_O, (II), are reported. Apart from a slight site occupany difference for the water mol­ecule of crystallization, compounds (I) and (II) are isostructural, featuring isolated tetra­hedral cations of copper(I) ions coordinated by two dmbpy ligands and centrosymmetric, octa­hedral anions of fluorinated early transition metals. The tetra­hedral environments of the copper complexes are distorted owing to the steric effects of the dmbpy ligands. The extended structures are built up through Coulombic inter­actions between cations and anions and π–π stacking inter­actions between heterochiral Δ- and Λ-[Cu(dmbpy)_2_]^+^ complexes. A comparison between the title compounds and other [Cu(dmbpy)_2_]^+^ compounds with monovalent and bivalent anions reveals a significant influence of the cation-to-anion ratio on the resulting crystal packing architectures, providing insights for future crystal design of distorted tetra­hedral copper compounds.

## Abstract 2

The syntheses and crystal structures of two bimetallic mol­ecular compounds, namely, bis­(6,6′-dimethyl-2,2′-bi­pyridine)­copper(I) hexa­fluorido­zirconate(IV) 1.134-hydrate, [Cu(dmbpy)_2_]_2_[ZrF_6_]·1.134H_2_O (dmbpy = 6,6′-dimethyl-2,2′-bipyridyl, C_12_H_12_N_2_), (I), and bis­(6,6′-dimethyl-2,2′-bi­pyridine)­copper(I) hexa­fluorido­hafnate(IV) 0.671-hydrate, [Cu(dmbpy)_2_]_2_[HfF_6_]·0.671H_2_O, (II), are reported. Apart from a slight site occupancy difference for the water mol­ecule of crystallization, compounds (I) and (II) are isostructural, featuring isolated tetra­hedral cations of copper(I) ions coordinated by two dmbpy ligands and centrosymmetric, octa­hedral anions of fluorinated early transition metals.

## Chemical context   

Copper(I) complexes with distorted tetra­hedral environments have been studied as catalytic active sites in electron-transfer reactions and are found in a number of proteins that contain copper (Vallee & Williams, 1968 ▸; Colman *et al.*, 1978 ▸; Adman *et al.*, 1978 ▸). The realization of significantly distorted tetra­hedral geometry requires sufficient steric hindrance between the ligands. The methyl groups of the 6,6′-dimethyl-2,2′-bipyridyl (C_12_H_12_N_2_; dmbpy) ligand create a large steric hindrance upon coordination, and, consequently, a common strategy to form distorted tetra­hedral complexes is to use dmbpy or its derivatives as ligands (McKenzie *et al.*, 1971 ▸; Burke *et al.*, 1980 ▸). Previously, compounds with distorted tetra­hedral [Cu(dmbpy)_2_]^+^ cations have been reported, namely [Cu(dmbpy)_2_]*X* (*X* = [BF_4_]^−^, [ClO_4_]^−^, [PF­_6_]^−^), [Cu(dmbpy)_2_][C_16_H_9_O_8_]·H_2_O (C_16_H_9_O_8_ = 2′,3,3′-tri­carb­oxy­biphenyl-2-carboxyl­ate) and [Cu(dmbpy)_2_]*X*_2_ (*X* = [BF_4_]^−^, [ClO_4_]^−^). (Burke *et al.*, 1980 ▸; Cui *et al.*, 2005 ▸; Itoh *et al.*, 2005 ▸; Mei *et al.*, 2011 ▸; Bozic-Weber *et al.*, 2012 ▸; Li *et al.*, 2017 ▸) Here, we report two structures with [*M*F_6_]^2−^ (*M* = Zr, Hf), which are the first known distorted tetra­hedral copper compounds with bivalent anions. 
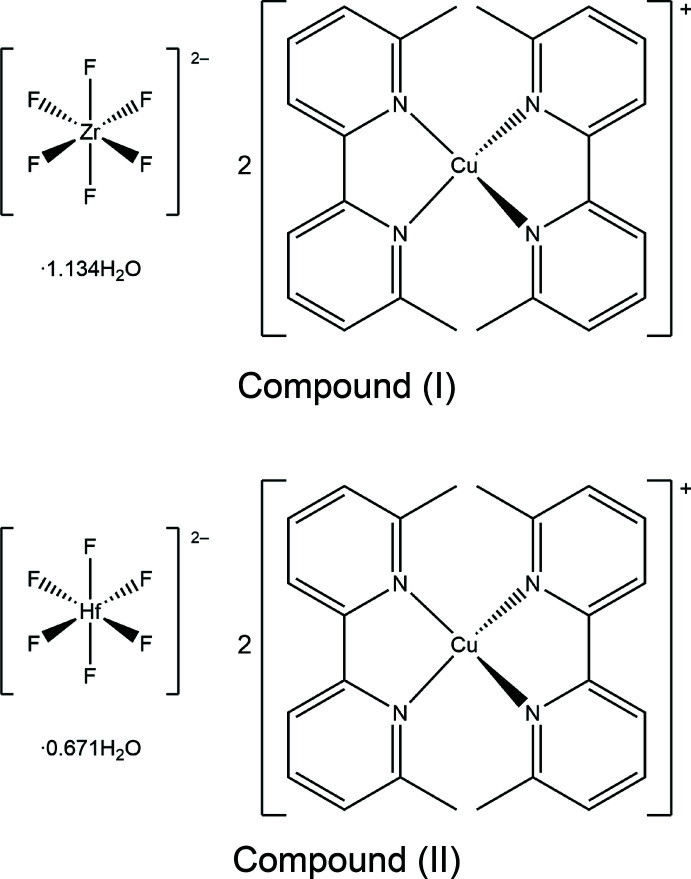


## Structural commentary   

**Figure 1:**
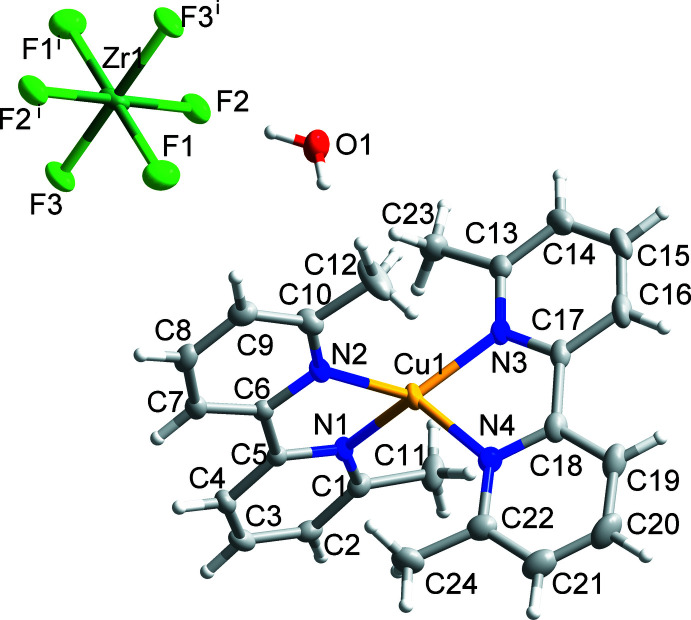
The mol­ecular structure of (I) showing 50% displacement ellipsoids. Symmetry code: (i) −*x*, 2 − *y*, 2 − *z*.

Compound (I) has the formula [Cu(dmbpy)_2_]_2_[ZrF_6_]·1.134H_2_O and crystallizes in the triclinic space group *P* 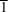 (Fig. 1 ▸). The structure of compound (I) features isolated tetra­hedral [Cu(dmbpy)_2_]^+^ cations and octa­hedral ZrF_6_^2−^ anions (Zr site symmetry 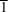 ). The coordination geometry of Cu1 and its donor N atoms deviates from an ideal tetra­hedron, as demonstrated by the 83.33 (10)° angle between the least squares planes containing Cu1 and each ligand (Table 1 ▸). To qu­antify the deviation from *T_d_* symmetry in [Cu(dmbpy)_2_]^+^ cations, the τ_4_’ parameter is employed and it gives a value of 0.66 for compound (I) (Okuniewski *et al.*, 2015 ▸). The distorted tetra­hedral geometry of [Cu(dmbpy)_2_]^+^ in compound (I) is consistent with other reported compounds containing [Cu(dmbpy)_2_]^+^ cations (Burke *et al.*, 1980 ▸; Cui *et al.*, 2005 ▸; Mei *et al.*, 2011 ▸; Bozic-Weber *et al.*, 2012 ▸). Moreover, the dmbpy ligands in (I) are non-planar and are slightly twisted on the 2,2′ carbon bond to give a dihedral angle of 8.68 (10)° between the N1/C1–C5 and N2/C6–C10 rings and 7.44 (11)° between the N3/C13–C17 and N4/C18–C22 rings. The distorted tetra­hedral environment and non-planar ligand geometry give the [Cu(dmbpy)_2_]^+^ cations a *C*_2_ symmetry, and enanti­omeric Δ- and Λ-[Cu(dmbpy)_2_]^+^ pairs are related across inversion centers. The octa­hedral coordination environment of Zr1 is slightly distorted, with Zr1—F bond lengths ranging from 1.9955 (13) to 2.0183 (12) Å (Table 1 ▸). The minor distortion of the ZrF_6_^2−^ anion may arise due to hydrogen-bonding inter­actions between water mol­ecules of crystallization and fluorine atoms on the *trans* position of the ZrF_6_^2−^ anions [see O1—H1*B*⋯F2 (Table 2 ▸)].

**Figure 2:**
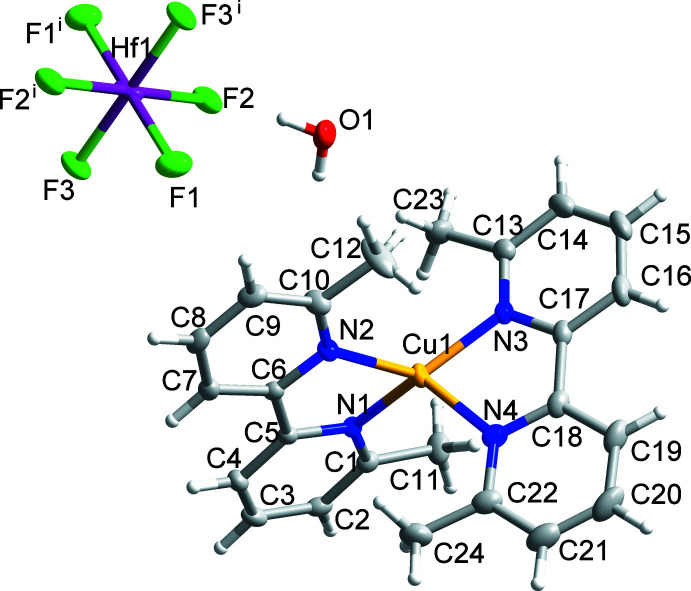
The mol­ecular structure of (II) showing 50% displacement ellipsoids. Symmetry code: (i) (i) −*x*, 2 − *y*, 2 − *z*.

Compound (II) has the formula [Cu(dmbpy)_2_]_2_[HfF_6_]·0.671H_2_O and crystallizes in the triclinic space group *P* 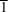 (Fig. 2 ▸). Compound (II) is isostructural to compound (I), therefore, the [Cu(dmbpy)_2_]^+^ cations also have *C*_2_ symmetry, with the angle between the least squares planes containing Cu1 and each ligand being 84.14 (8)° (Table 3 ▸) and the τ_4_’ parameter being 0.66, and the dmbpy ligands are slightly twisted on the 2,2′ carbon bond to give an angle of 9.69 (7)° between the N1/C1–C5 and N2/C6–C10 rings and 7.97 (8)° between the N3/C13–C17 and N4/C18–C22 rings. Moreover, the octa­hedral coordination environment of Hf1 is also slightly distorted, with Hf1—F bond lengths ranging from 1.9945 (10) to 2.0111 (11) Å. Like in compound (I), hydrogen-bonding inter­actions are present between the water mol­ecule of crystallization and fluorine atoms on the *trans* position of HfF_6_^2−^ anions, but the geometry of the hydrogen bond is slightly different from that in compound (I) [see O1—H1*B*⋯F2 (Table 4 ▸)].

## Supra­molecular features   

**Figure 3:**
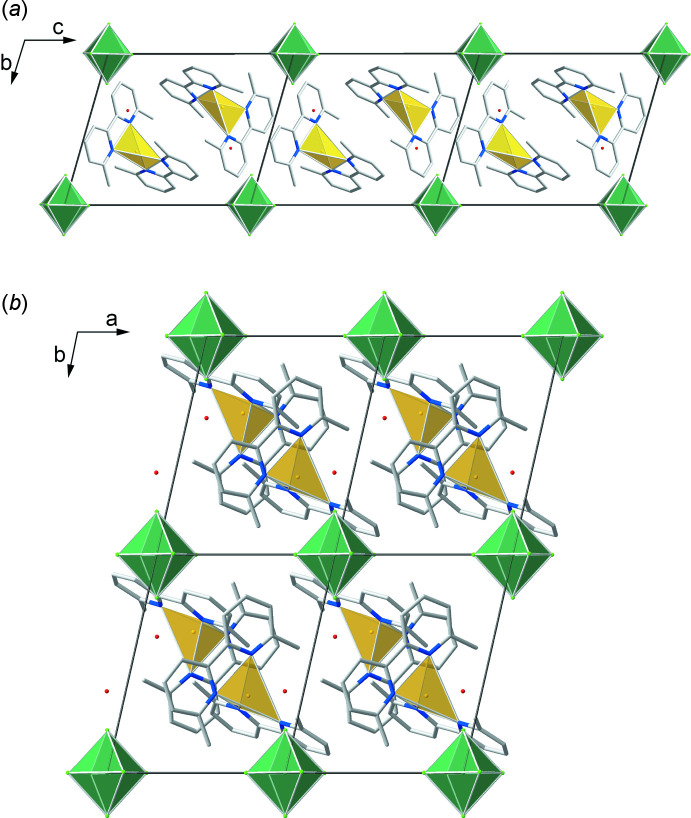
The packing for (I) viewed (*a*) down [100] and (*b*) down [001], with the copper and zirconium coordination environments represented by yellow/orange and green polyhedra, respectively.

In the extended structures of compounds (I) and (II), the [Cu(dmbpy)_2_]^+^ cations and octa­hedral *M*F_6_^2−^ anions are closely packed *via* Coulombic inter­actions (Fig. 3 ▸). The Δ/Λ-[Cu(dmbpy)_2_]^+^ cations stack into racemic pairs along the *c-*axis direction *via* a heterochiral face-to-face π–π inter­action between the N1/C1–C5 and N2/C6–C10 rings with an inter­planar angle of 0°, inter­planar distances of 3.347 and 3.355 Å, and centroid–centroid distances (*d*_py–py_) of 3.6967 (12) and 3.7016 (8) Å, for compounds (I) and (II), respectively (Tables 5 ▸ and 6 ▸). Next, Δ/Λ-[Cu(dmbpy)_2_]^+^ pairs pack into racemic chains along the *c-*axis direction with heterochiral parallel displaced π–π inter­actions between the N3/C13–C17 and N4/C18–C22 rings with an inter­planar angle of 0°, inter­planar distances of 3.708 and 3.678 Å, and centroid–centroid distances (*d*_py–py_) of 5.3726 (13) and 5.3777 (11) Å, for compounds (I) and (II), respectively. The *M*F_6_^2−^ anions with hydrogen-bonded water mol­ecules are inter­laced between the racemic chains to form the extended three-dimensional structure. Compared to other mol­ecular compounds with *M*F_6_^2−^ anions in an extended and complicated hydrogen network (Gautier *et al.*, 2012 ▸; Nisbet *et al.*, 2020 ▸, 2021 ▸), the *M*F_6_^2−^ anions in (I) and (II) experience less distortion because the hydrogen-bonding contacts are less extensive and only occur along the same axis due to the site symmetry of hydrogen-bonding inter­actions (Kunz & Brown, 1995 ▸; Halasyamani, 2004 ▸).

## Database survey   

A survey of compounds related to compounds (I) and (II) reported in the Cambridge Structural Database (CSD version 2020.1 from April 2020; Groom *et al.*, 2016 ▸) produced four other compounds based on [Cu(dmbpy)_2_]^+^ complexes: [Cu(dmbpy)_2_][BF­_4_] (CSD refcode: MPYRCU; Burke *et al.*, 1980 ▸), [Cu(dmbpy)_2_][PF­_6_] (REFSUS; Bozic-Weber *et al.*, 2012 ▸), [Cu(dmbpy)_2_][ClO­_4_] (FAXLAS; Cui *et al.*, 2005 ▸), and [Cu(dmbpy)_2_][C_16_H_9_O_8_]·H_2_O (C_16_H_9_O_8_ = 2′,3,3′-tri­carb­oxy­biphenyl-2-carboxyl­ate) (ABIYER; Mei *et al.*, 2011 ▸). All these structures have distorted tetra­hedral [Cu(dmbpy)_2_]^+^ cations with *C*_2_ symmetry, with a range of the angle between the least-squares planes containing the metal ion and each ligand being from 75.06 to 86.74°. Moreover, τ_4_’ parameters for these structures range from 0.70 to 0.74, whereas for both compound (I) and (II) the parameter is 0.66 (Okuniewski *et al.*, 2015 ▸).

Unlike compound (I) and (II), which have bivalent anions *M*F_6_^2−^, the compounds reported in the CSD are charge-balanced by monovalent anions and display two different types of packing architectures distinct from those of the title compounds: [Cu(dmbpy)_2_][BF­_4_], [Cu(dmbpy)_2_][PF­_6_], and [Cu(dmbpy)_2_][ClO­_4_] are isostructural, crystallizing in space group *P2_1_/c*. Compared to compounds (I) and (II), the ratio of cations-to-anions is smaller in these monovalent-anion compounds. Instead of racemic chains, homochiral chains are observed with homochiral displaced π–π inter­actions between the ligands with an inter­planar angle of around 30°. No local or extended hydrogen-bond networks are observed because these structures do not contain water mol­ecules of crystallization.

Another type of packing architecture is found in [Cu(dmbpy)_2_][C_16_H_9_O_8_]·H_2_O, which crystallizes in space group *P* 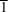 . Unlike the aforementioned five compounds with [Cu(dmbpy)_2_]^+^ cations, π–π inter­actions in the compound [Cu(dmbpy)_2_][C_16_H_9_O_8_]·H_2_O are dominant between [Cu(dmbpy)_2_]^+^ cations and [C_16_H_9_O_8_]^−^ anions instead of between [Cu(dmbpy)_2_]^+^ cations. In this compound, the [Cu(dmbpy)_2_]^+^ cations and [C_16_H_9_O_8_]^−^ anions are packed into charge-neutral chains *via* Coulombic inter­actions and π–π inter­actions along *c* axis and inversion centers are present between the chains. Additionally, the [C_16_H_9_O_8_]^−^ anions and free water mol­ecules generate a three-dimensional network *via* O—H⋯O hydrogen bonding inter­actions, resulting in a different architecture.

## Synthesis and crystallization   

The compounds reported here were synthesized by the hydro­thermal pouch method (Harrison *et al.*, 1993 ▸). In each reaction, reagents were heat-sealed in Teflon pouches. Groups of six pouches were then placed into a 125 ml Parr autoclave with 45 ml of distilled water as backfill. The autoclave was heated at a rate of 5 K min^−1^ to 423 K and held at 423 K for 24 h. The autoclaves were allowed to cool to room temperature at a rate of 6 K h^−1^. Orangish red solid products were recovered by vacuum filtration with a moderate yield. Compound (I) was synthesized in a pouch containing 0.4195 mmol of CuO, 0.4195 mmol of ZrO_2_, 0.835 mmol of 6,6′-dimethyl-2,2′-bipyridyl, 0.15 ml (4.14 mmol) of HF (aq) (48%), and 0.1 ml (5.5 mmol) of deionized H_2_O. Compound (II) was synthesized in a pouch containing 0.4195 mmol of CuO, 0.4195 mmol of HfO_2_, 0.835 mmol of 6,6′-dimethyl-2,2′-bipyridyl, 0.05 ml (1.38 mmol) of HF (aq) (48%), and 0.2 ml (11 mmol) of deionized H_2_O.

## Refinement   

Crystal data, data collection and structure refinement details are summarized in Table 7 ▸. Hydrogen-atom positions were assigned from difference map peaks with the exception of the C—H hydrogen atoms of dmbpy, which were constrained to ride at distances of 0.95 Å from the associated C atoms with *U*_iso_(H) = 1.2*U*_eq_(C) within *OLEX2* (Dolomanov *et al.*, 2009 ▸). The water occupancies in both structures are refined freely. Four reflections showing very poor agreement were omitted from the final refinement for compound (I).

## Supplementary Material

Crystal structure: contains datablock(s) I, II. DOI: 10.1107/S2056989021007295/hb7977sup1.cif

Structure factors: contains datablock(s) I. DOI: 10.1107/S2056989021007295/hb7977Isup2.hkl

Structure factors: contains datablock(s) II. DOI: 10.1107/S2056989021007295/hb7977IIsup3.hkl

CCDC references: 2096336, 2096335

Additional supporting information:  crystallographic information; 3D view; checkCIF report

## Figures and Tables

**Table 1:** Selected geometric parameters (Å, °) for (I)

Cu1—N1	2.0208 (16)	Zr1—F1	2.0113 (15)
Cu1—N2	2.0348 (17)	Zr1—F2	2.0183 (12)
Cu1—N3	2.0123 (17)	Zr1—F3	1.9955 (13)
Cu1—N4	2.0616 (18)		
			
N1—Cu1—N2	81.40 (7)	N3—Cu1—N1	136.29 (7)
N1—Cu1—N4	116.24 (7)	N3—Cu1—N2	126.16 (7)
N2—Cu1—N4	120.45 (7)	N3—Cu1—N4	81.05 (7)

**Table 2:** Hydrogen-bond geometry (Å, °) for (I)

*D*—H⋯*A*	*D*—H	H⋯*A*	*D*⋯*A*	*D*—H⋯*A*
O1—H1*B*⋯F2	0.87	1.47	2.337 (4)	177

**Table 3:** Selected geometric parameters (Å, °) for (II)

Cu1—N1	2.0229 (12)	Hf1—F1	2.0111 (11)
Cu1—N2	2.0414 (12)	Hf1—F2	2.0033 (9)
Cu1—N3	2.0121 (12)	Hf1—F3	1.9945 (10)
Cu1—N4	2.0659 (13)		
			
N1—Cu1—N2	81.22 (5)	N3—Cu1—N1	136.20 (5)
N1—Cu1—N4	116.52 (5)	N3—Cu1—N2	126.39 (5)
N2—Cu1—N4	120.35 (5)	N3—Cu1—N4	80.94 (5)

**Table 4:** Hydrogen-bond geometry (Å, °) for (II)

*D*—H⋯*A*	*D*—H	H⋯*A*	*D*⋯*A*	*D*—H⋯*A*
O1B—H1*B*⋯F2	0.87	1.50	2.328 (4)	156

**Table 5:** Aromatic π–π stacking inter­actions (Å, °) in (I)

Description	type	*d*_py–py_	inter­planar angle	inter­planar distance
Heterochiral	face-to-face	3.6967 (12)	0	3.347
Heterochiral	parallel displaced	5.3726 (13)	0	3.708

**Table 6:** Aromatic π–π stacking inter­actions (Å, °) in (II)

Description	type	*d*_py–py_	inter­planar angle	inter­planar distance
Heterochiral	face-to-face	3.7016 (8)	0	3.355
Heterochiral	parallel displaced	5.3777 (11)	0	3.678

**Table 7:** Experimental details

	(I)	(II)
Crystal data		
Chemical formula	[Cu(C_12_H_12_N_2_)_2_]_2_[ZrF_6_]·1.134H_2_O	[Cu(C_12_H_12_N_2_)_2_]_2_[HfF_6_]·0.671H_2_O
*M*_r_	1089.61	1168.58
Crystal system, space group	Triclinic, *P*\overline{1}	Triclinic, *P*\overline{1}
Temperature (K)	100	100
*a*, *b*, *c* (Å)	8.6219 (3), 10.8064 (3), 12.9992 (4)	8.5737 (1), 10.7967 (2), 13.0183 (2)
α, β, γ (°)	103.078 (2), 104.013 (3), 98.863 (2)	103.273 (1), 103.662 (1), 98.785 (1)
*V* (Å^3^)	1116.33 (6)	1112.07 (3)
*Z*	1	1
Radiation type	Mo *K*α	Mo *K*α
μ (mm^−1^)	1.25	3.35
Crystal size (mm)	0.98 × 0.13 × 0.05	0.3 × 0.17 × 0.08

Data collection		
Diffractometer	Rigaku Saturn724+ (2x2 bin mode)	Rigaku Saturn724+ (2x2 bin mode)
Absorption correction	Gaussian (*CrysAlis PRO*; Rigaku OD, 2020 ▸)	Gaussian (*CrysAlis PRO*; Rigaku OD, 2020 ▸)
*T*_min_, *T*_max_	0.376, 1.000	0.433, 1.000
No. of measured, independent and observed [*I* > 2σ(*I*)] reflections	16573, 5673, 4588	40796, 8003, 7235
*R*_int_	0.039	0.032
(sin θ/λ)_max_ (Å^−1^)	0.722	0.784

Refinement		
*R*[*F* ^2^ > 2σ(*F* ^2^)], *wR*(*F* ^2^), *S*	0.035, 0.088, 1.07	0.023, 0.056, 1.08
No. of reflections	5673	8003
No. of parameters	312	312
H-atom treatment	H-atom parameters constrained	H-atom parameters constrained
Δρ_max_, Δρ_min_ (e Å^−3^)	0.59, −0.54	0.48, −0.73

Computer programs: *CrysAlis PRO* (Rigaku OD, 2020 ▸), *SHELXT2014/5* (Sheldrick, 2015*a* ▸), *SHELXL* (Sheldrick, 2015*b* ▸), and *OLEX2* (Dolomanov *et al.*, 2009 ▸).

## supplementary crystallographic information

### Bis[bis(6,6'-dimethyl-2,2'-bipyridine)copper(I)] hexafluoridozirconate(IV) 1.134-hydrate (I) Crystal data

**Table:**

[Cu(C_12_H_12_N_2_)_2_]_2_[ZrF_6_]·1.134H_2_O	*Z* = 1
*M**_r_* = 1089.61	*F*(000) = 555
Triclinic, *P*1	*D*_x_ = 1.621 Mg m^−^^3^
*a* = 8.6219 (3) Å	Mo *K*α radiation, λ = 0.71073 Å
*b* = 10.8064 (3) Å	Cell parameters from 10429 reflections
*c* = 12.9992 (4) Å	θ = 2.2–30.6°
α = 103.078 (2)°	µ = 1.25 mm^−^^1^
β = 104.013 (3)°	*T* = 100 K
γ = 98.863 (2)°	Needle, clear orangish red
*V* = 1116.33 (6) Å^3^	0.98 × 0.13 × 0.05 mm

### Bis[bis(6,6'-dimethyl-2,2'-bipyridine)copper(I)] hexafluoridozirconate(IV) 1.134-hydrate (I) Data collection

**Table:**

Rigaku Saturn724+ (2x2 bin mode) diffractometer	5673 independent reflections
Radiation source: Rotating Anode, Rotating Anode	4588 reflections with *I* > 2σ(*I*)
Confocal monochromator	*R*_int_ = 0.039
Detector resolution: 28.5714 pixels mm^-1^	θ_max_ = 30.9°, θ_min_ = 2.0°
profile data from ω–scans	*h* = −11→11
Absorption correction: gaussian (CrysAlisPro; Rigaku OD, 2020)	*k* = −14→15
*T*_min_ = 0.376, *T*_max_ = 1.000	*l* = −16→18
16573 measured reflections	

### Bis[bis(6,6'-dimethyl-2,2'-bipyridine)copper(I)] hexafluoridozirconate(IV) 1.134-hydrate (I) Refinement

**Table:**

Refinement on *F*^2^	Primary atom site location: dual
Least-squares matrix: full	Hydrogen site location: mixed
*R*[*F*^2^ > 2σ(*F*^2^)] = 0.035	H-atom parameters constrained
*wR*(*F*^2^) = 0.088	*w* = 1/[σ^2^(*F*_o_^2^) + (0.0397*P*)^2^ + 0.3898*P*] where *P* = (*F*_o_^2^ + 2*F*_c_^2^)/3
*S* = 1.07	(Δ/σ)_max_ = 0.001
5673 reflections	Δρ_max_ = 0.59 e Å^−^^3^
312 parameters	Δρ_min_ = −0.54 e Å^−^^3^
0 restraints	

### Bis[bis(6,6'-dimethyl-2,2'-bipyridine)copper(I)] hexafluoridozirconate(IV) 1.134-hydrate (I) Special details

**Table:**

Geometry. All esds (except the esd in the dihedral angle between two l.s. planes) are estimated using the full covariance matrix. The cell esds are taken into account individually in the estimation of esds in distances, angles and torsion angles; correlations between esds in cell parameters are only used when they are defined by crystal symmetry. An approximate (isotropic) treatment of cell esds is used for estimating esds involving l.s. planes.

### Bis[bis(6,6'-dimethyl-2,2'-bipyridine)copper(I)] hexafluoridozirconate(IV) 1.134-hydrate (I) Fractional atomic coordinates and isotropic or equivalent isotropic displacement parameters (Å^2^)

**Table:**

	*x*	*y*	*z*	*U*_iso_*/*U*_eq_	Occ. (<1)
Cu1	0.30548 (3)	0.35420 (2)	0.69756 (2)	0.02018 (8)	
N1	0.4953 (2)	0.36543 (16)	0.82845 (13)	0.0174 (3)	
C1	0.5623 (3)	0.26739 (19)	0.85293 (17)	0.0207 (4)	
N2	0.3424 (2)	0.54652 (16)	0.77601 (14)	0.0195 (3)	
C2	0.7120 (3)	0.2905 (2)	0.93235 (17)	0.0212 (4)	
H2	0.756689	0.219858	0.948974	0.025*	
N3	0.0873 (2)	0.23159 (15)	0.61598 (14)	0.0190 (3)	
C3	0.7947 (3)	0.4174 (2)	0.98671 (17)	0.0230 (4)	
H3	0.898128	0.435022	1.040244	0.028*	
N4	0.3423 (2)	0.29725 (16)	0.54450 (14)	0.0211 (4)	
C4	0.7257 (2)	0.5187 (2)	0.96256 (17)	0.0210 (4)	
H4	0.780962	0.606656	0.999235	0.025*	
C5	0.5745 (2)	0.48980 (18)	0.88392 (16)	0.0160 (4)	
C6	0.4869 (2)	0.59134 (18)	0.85644 (16)	0.0166 (4)	
C7	0.5450 (3)	0.72317 (19)	0.91116 (17)	0.0202 (4)	
H7	0.649798	0.753655	0.964086	0.024*	
C8	0.4472 (3)	0.8091 (2)	0.88699 (18)	0.0237 (4)	
H8	0.484553	0.899537	0.923175	0.028*	
C9	0.2955 (3)	0.7627 (2)	0.81024 (19)	0.0251 (4)	
H9	0.224458	0.819860	0.796195	0.030*	
C10	0.2476 (3)	0.6306 (2)	0.75341 (19)	0.0247 (4)	
C11	0.4653 (3)	0.1315 (2)	0.7923 (2)	0.0325 (5)	
H11A	0.374488	0.110516	0.822909	0.049*	
H11B	0.536537	0.069734	0.800414	0.049*	
H11C	0.421624	0.125560	0.713932	0.049*	
C12	0.0897 (3)	0.5747 (3)	0.6624 (3)	0.0505 (8)	
H12A	0.113803	0.542757	0.592501	0.076*	
H12B	0.027094	0.642511	0.656716	0.076*	
H12C	0.025358	0.502569	0.678793	0.076*	
C13	−0.0357 (3)	0.20374 (19)	0.65956 (18)	0.0222 (4)	
C14	−0.1902 (3)	0.1331 (2)	0.5926 (2)	0.0299 (5)	
H14	−0.276649	0.114742	0.624100	0.036*	
C15	−0.2168 (3)	0.0899 (2)	0.4802 (2)	0.0331 (6)	
H15	−0.322268	0.042926	0.433723	0.040*	
C16	−0.0896 (3)	0.1152 (2)	0.43557 (19)	0.0286 (5)	
H16	−0.105281	0.084169	0.358515	0.034*	
C17	0.0622 (3)	0.18722 (18)	0.50567 (17)	0.0209 (4)	
C18	0.2076 (3)	0.2182 (2)	0.46677 (17)	0.0228 (4)	
C19	0.2083 (3)	0.1682 (2)	0.35859 (18)	0.0297 (5)	
H19	0.112010	0.114338	0.304771	0.036*	
C20	0.3513 (4)	0.1981 (2)	0.3308 (2)	0.0356 (6)	
H20	0.354850	0.164937	0.257431	0.043*	
C21	0.4888 (3)	0.2767 (2)	0.4105 (2)	0.0344 (5)	
H21	0.588640	0.296366	0.392590	0.041*	
C22	0.4816 (3)	0.3270 (2)	0.51691 (19)	0.0260 (5)	
C23	0.0017 (3)	0.2514 (2)	0.78169 (19)	0.0308 (5)	
H23A	0.106137	0.232552	0.816790	0.046*	
H23B	−0.085860	0.207350	0.805470	0.046*	
H23C	0.009325	0.345593	0.803131	0.046*	
C24	0.6265 (3)	0.4151 (2)	0.6065 (2)	0.0325 (5)	
H24A	0.593595	0.492033	0.643469	0.049*	
H24B	0.713290	0.442318	0.574399	0.049*	
H24C	0.667181	0.368394	0.660221	0.049*	
Zr1	0.000000	1.000000	1.000000	0.02476 (9)	
F1	0.09081 (18)	0.99518 (14)	0.87082 (13)	0.0384 (3)	
F2	−0.05473 (16)	0.80350 (12)	0.95926 (12)	0.0322 (3)	
F3	0.22027 (15)	1.00042 (12)	1.09456 (13)	0.0346 (3)	
O1	−0.1062 (4)	0.6247 (3)	0.8061 (3)	0.0369 (11)	0.567 (6)
H1A	−0.045474	0.641036	0.764081	0.055*	0.567 (6)
H1B	−0.087438	0.692874	0.861724	0.055*	0.567 (6)

### Bis[bis(6,6'-dimethyl-2,2'-bipyridine)copper(I)] hexafluoridozirconate(IV) 1.134-hydrate (I) Atomic displacement parameters (Å^2^)

**Table:**

	*U*^11^	*U*^22^	*U*^33^	*U*^12^	*U*^13^	*U*^23^
Cu1	0.02111 (14)	0.01747 (13)	0.01614 (13)	0.00081 (10)	−0.00092 (10)	0.00225 (9)
N1	0.0200 (8)	0.0173 (8)	0.0128 (8)	0.0026 (7)	0.0022 (6)	0.0033 (6)
C1	0.0253 (10)	0.0205 (10)	0.0184 (10)	0.0078 (8)	0.0073 (8)	0.0065 (8)
N2	0.0193 (8)	0.0167 (8)	0.0198 (9)	0.0029 (7)	0.0016 (7)	0.0051 (6)
C2	0.0232 (10)	0.0255 (10)	0.0205 (10)	0.0114 (8)	0.0095 (8)	0.0100 (8)
N3	0.0226 (9)	0.0142 (8)	0.0175 (8)	0.0038 (7)	0.0022 (7)	0.0029 (6)
C3	0.0168 (9)	0.0325 (11)	0.0204 (10)	0.0052 (8)	0.0036 (8)	0.0109 (9)
N4	0.0251 (9)	0.0206 (8)	0.0191 (9)	0.0049 (7)	0.0066 (7)	0.0082 (7)
C4	0.0180 (10)	0.0206 (10)	0.0203 (10)	0.0007 (8)	0.0023 (8)	0.0039 (8)
C5	0.0165 (9)	0.0167 (9)	0.0148 (9)	0.0033 (7)	0.0051 (7)	0.0040 (7)
C6	0.0169 (9)	0.0174 (9)	0.0160 (9)	0.0031 (7)	0.0058 (7)	0.0048 (7)
C7	0.0211 (10)	0.0188 (9)	0.0194 (10)	0.0008 (8)	0.0064 (8)	0.0043 (8)
C8	0.0334 (12)	0.0168 (9)	0.0224 (11)	0.0039 (9)	0.0123 (9)	0.0048 (8)
C9	0.0289 (11)	0.0222 (10)	0.0311 (12)	0.0102 (9)	0.0128 (9)	0.0131 (9)
C10	0.0223 (10)	0.0231 (10)	0.0293 (12)	0.0045 (8)	0.0037 (9)	0.0125 (9)
C11	0.0456 (14)	0.0190 (10)	0.0265 (12)	0.0104 (10)	−0.0010 (10)	0.0033 (9)
C12	0.0333 (14)	0.0281 (13)	0.073 (2)	0.0029 (11)	−0.0190 (14)	0.0192 (13)
C13	0.0230 (10)	0.0173 (9)	0.0245 (11)	0.0053 (8)	0.0055 (8)	0.0031 (8)
C14	0.0234 (11)	0.0204 (10)	0.0428 (14)	0.0019 (9)	0.0087 (10)	0.0057 (10)
C15	0.0247 (12)	0.0217 (11)	0.0382 (14)	0.0006 (9)	−0.0059 (10)	−0.0017 (10)
C16	0.0335 (12)	0.0195 (10)	0.0227 (11)	0.0050 (9)	−0.0054 (9)	0.0007 (8)
C17	0.0276 (11)	0.0148 (9)	0.0170 (10)	0.0062 (8)	−0.0007 (8)	0.0044 (7)
C18	0.0315 (11)	0.0196 (10)	0.0166 (10)	0.0070 (9)	0.0021 (8)	0.0077 (8)
C19	0.0473 (14)	0.0226 (11)	0.0180 (11)	0.0090 (10)	0.0056 (10)	0.0065 (9)
C20	0.0602 (17)	0.0319 (12)	0.0245 (12)	0.0163 (12)	0.0222 (12)	0.0118 (10)
C21	0.0465 (15)	0.0345 (13)	0.0357 (14)	0.0170 (11)	0.0239 (12)	0.0178 (11)
C22	0.0304 (11)	0.0239 (10)	0.0310 (12)	0.0107 (9)	0.0132 (9)	0.0137 (9)
C23	0.0309 (12)	0.0341 (12)	0.0278 (12)	0.0045 (10)	0.0122 (10)	0.0068 (10)
C24	0.0258 (12)	0.0367 (13)	0.0384 (14)	0.0034 (10)	0.0134 (10)	0.0147 (11)
Zr1	0.01462 (14)	0.02394 (15)	0.03837 (19)	0.00405 (11)	0.00458 (12)	0.01715 (13)
F1	0.0390 (8)	0.0355 (8)	0.0507 (9)	0.0129 (6)	0.0198 (7)	0.0209 (7)
F2	0.0256 (7)	0.0188 (6)	0.0479 (9)	0.0022 (5)	0.0034 (6)	0.0104 (6)
F3	0.0207 (6)	0.0230 (6)	0.0511 (9)	0.0052 (5)	−0.0042 (6)	0.0081 (6)
O1	0.041 (2)	0.0389 (19)	0.0244 (17)	0.0009 (14)	0.0117 (14)	0.0001 (13)

### Bis[bis(6,6'-dimethyl-2,2'-bipyridine)copper(I)] hexafluoridozirconate(IV) 1.134-hydrate (I) Geometric parameters (Å, º)

**Table:**

Cu1—N1	2.0208 (16)	C12—H12B	0.9800
Cu1—N2	2.0348 (17)	C12—H12C	0.9800
Cu1—N3	2.0123 (17)	C13—C14	1.392 (3)
Cu1—N4	2.0616 (18)	C13—C23	1.490 (3)
N1—C1	1.346 (3)	C14—H14	0.9500
N1—C5	1.354 (2)	C14—C15	1.379 (4)
C1—C2	1.390 (3)	C15—H15	0.9500
C1—C11	1.502 (3)	C15—C16	1.379 (4)
N2—C6	1.355 (2)	C16—H16	0.9500
N2—C10	1.346 (3)	C16—C17	1.392 (3)
C2—H2	0.9500	C17—C18	1.481 (3)
C2—C3	1.380 (3)	C18—C19	1.390 (3)
N3—C13	1.344 (3)	C19—H19	0.9500
N3—C17	1.356 (3)	C19—C20	1.379 (4)
C3—H3	0.9500	C20—H20	0.9500
C3—C4	1.385 (3)	C20—C21	1.377 (4)
N4—C18	1.355 (3)	C21—H21	0.9500
N4—C22	1.347 (3)	C21—C22	1.388 (3)
C4—H4	0.9500	C22—C24	1.500 (3)
C4—C5	1.388 (3)	C23—H23A	0.9800
C5—C6	1.485 (3)	C23—H23B	0.9800
C6—C7	1.391 (3)	C23—H23C	0.9800
C7—H7	0.9500	C24—H24A	0.9800
C7—C8	1.384 (3)	C24—H24B	0.9800
C8—H8	0.9500	C24—H24C	0.9800
C8—C9	1.378 (3)	Zr1—F1	2.0113 (15)
C9—H9	0.9500	Zr1—F1^i^	2.0113 (15)
C9—C10	1.396 (3)	Zr1—F2^i^	2.0183 (12)
C10—C12	1.503 (3)	Zr1—F2	2.0183 (12)
C11—H11A	0.9800	Zr1—F3	1.9955 (13)
C11—H11B	0.9800	Zr1—F3^i^	1.9955 (13)
C11—H11C	0.9800	O1—H1A	0.8699
C12—H12A	0.9800	O1—H1B	0.8703
			
N1—Cu1—N2	81.40 (7)	H12B—C12—H12C	109.5
N1—Cu1—N4	116.24 (7)	N3—C13—C14	120.9 (2)
N2—Cu1—N4	120.45 (7)	N3—C13—C23	116.93 (18)
N3—Cu1—N1	136.29 (7)	C14—C13—C23	122.2 (2)
N3—Cu1—N2	126.16 (7)	C13—C14—H14	120.2
N3—Cu1—N4	81.05 (7)	C15—C14—C13	119.6 (2)
C1—N1—Cu1	127.37 (14)	C15—C14—H14	120.2
C1—N1—C5	119.06 (17)	C14—C15—H15	120.2
C5—N1—Cu1	112.61 (13)	C14—C15—C16	119.7 (2)
N1—C1—C2	121.76 (18)	C16—C15—H15	120.2
N1—C1—C11	116.67 (19)	C15—C16—H16	120.7
C2—C1—C11	121.55 (19)	C15—C16—C17	118.7 (2)
C6—N2—Cu1	112.50 (13)	C17—C16—H16	120.7
C10—N2—Cu1	128.32 (14)	N3—C17—C16	121.5 (2)
C10—N2—C6	119.07 (17)	N3—C17—C18	115.35 (18)
C1—C2—H2	120.5	C16—C17—C18	123.1 (2)
C3—C2—C1	119.10 (19)	N4—C18—C17	115.47 (18)
C3—C2—H2	120.5	N4—C18—C19	121.7 (2)
C13—N3—Cu1	125.56 (14)	C19—C18—C17	122.8 (2)
C13—N3—C17	119.66 (18)	C18—C19—H19	120.6
C17—N3—Cu1	114.36 (14)	C20—C19—C18	118.8 (2)
C2—C3—H3	120.3	C20—C19—H19	120.6
C2—C3—C4	119.43 (19)	C19—C20—H20	120.3
C4—C3—H3	120.3	C21—C20—C19	119.4 (2)
C18—N4—Cu1	113.01 (14)	C21—C20—H20	120.3
C22—N4—Cu1	127.62 (15)	C20—C21—H21	120.0
C22—N4—C18	119.34 (19)	C20—C21—C22	119.9 (2)
C3—C4—H4	120.5	C22—C21—H21	120.0
C3—C4—C5	118.94 (18)	N4—C22—C21	120.8 (2)
C5—C4—H4	120.5	N4—C22—C24	116.8 (2)
N1—C5—C4	121.67 (18)	C21—C22—C24	122.3 (2)
N1—C5—C6	115.42 (17)	C13—C23—H23A	109.5
C4—C5—C6	122.90 (17)	C13—C23—H23B	109.5
N2—C6—C5	115.28 (16)	C13—C23—H23C	109.5
N2—C6—C7	121.72 (18)	H23A—C23—H23B	109.5
C7—C6—C5	122.97 (18)	H23A—C23—H23C	109.5
C6—C7—H7	120.6	H23B—C23—H23C	109.5
C8—C7—C6	118.70 (19)	C22—C24—H24A	109.5
C8—C7—H7	120.6	C22—C24—H24B	109.5
C7—C8—H8	120.1	C22—C24—H24C	109.5
C9—C8—C7	119.71 (19)	H24A—C24—H24B	109.5
C9—C8—H8	120.1	H24A—C24—H24C	109.5
C8—C9—H9	120.5	H24B—C24—H24C	109.5
C8—C9—C10	119.0 (2)	F1^i^—Zr1—F1	180.00 (9)
C10—C9—H9	120.5	F1—Zr1—F2^i^	89.69 (6)
N2—C10—C9	121.56 (19)	F1—Zr1—F2	90.31 (6)
N2—C10—C12	116.3 (2)	F1^i^—Zr1—F2	89.69 (6)
C9—C10—C12	122.1 (2)	F1^i^—Zr1—F2^i^	90.31 (6)
C1—C11—H11A	109.5	F2—Zr1—F2^i^	180.0
C1—C11—H11B	109.5	F3^i^—Zr1—F1	89.90 (6)
C1—C11—H11C	109.5	F3—Zr1—F1^i^	89.90 (6)
H11A—C11—H11B	109.5	F3—Zr1—F1	90.10 (6)
H11A—C11—H11C	109.5	F3^i^—Zr1—F1^i^	90.10 (6)
H11B—C11—H11C	109.5	F3^i^—Zr1—F2^i^	89.43 (5)
C10—C12—H12A	109.5	F3—Zr1—F2	89.43 (5)
C10—C12—H12B	109.5	F3—Zr1—F2^i^	90.57 (5)
C10—C12—H12C	109.5	F3^i^—Zr1—F2	90.57 (5)
H12A—C12—H12B	109.5	F3^i^—Zr1—F3	180.0
H12A—C12—H12C	109.5	H1A—O1—H1B	109.5
			
Cu1—N1—C1—C2	166.88 (15)	C5—N1—C1—C2	−1.0 (3)
Cu1—N1—C1—C11	−14.7 (3)	C5—N1—C1—C11	177.37 (19)
Cu1—N1—C5—C4	−167.35 (15)	C5—C6—C7—C8	−173.85 (19)
Cu1—N1—C5—C6	14.0 (2)	C6—N2—C10—C9	0.5 (3)
Cu1—N2—C6—C5	−9.8 (2)	C6—N2—C10—C12	179.4 (2)
Cu1—N2—C6—C7	172.18 (15)	C6—C7—C8—C9	0.2 (3)
Cu1—N2—C10—C9	−175.40 (16)	C7—C8—C9—C10	−3.8 (3)
Cu1—N2—C10—C12	3.4 (3)	C8—C9—C10—N2	3.6 (3)
Cu1—N3—C13—C14	170.26 (16)	C8—C9—C10—C12	−175.2 (2)
Cu1—N3—C13—C23	−10.0 (3)	C10—N2—C6—C5	173.65 (18)
Cu1—N3—C17—C16	−171.70 (16)	C10—N2—C6—C7	−4.4 (3)
Cu1—N3—C17—C18	9.7 (2)	C11—C1—C2—C3	−179.0 (2)
Cu1—N4—C18—C17	−0.4 (2)	C13—N3—C17—C16	1.3 (3)
Cu1—N4—C18—C19	−179.26 (16)	C13—N3—C17—C18	−177.34 (18)
Cu1—N4—C22—C21	177.41 (16)	C13—C14—C15—C16	1.1 (4)
Cu1—N4—C22—C24	−1.9 (3)	C14—C15—C16—C17	−1.7 (3)
N1—C1—C2—C3	−0.7 (3)	C15—C16—C17—N3	0.5 (3)
N1—C5—C6—N2	−2.7 (3)	C15—C16—C17—C18	179.0 (2)
N1—C5—C6—C7	175.24 (18)	C16—C17—C18—N4	175.31 (19)
C1—N1—C5—C4	2.3 (3)	C16—C17—C18—C19	−5.9 (3)
C1—N1—C5—C6	−176.43 (18)	C17—N3—C13—C14	−1.9 (3)
C1—C2—C3—C4	1.2 (3)	C17—N3—C13—C23	177.87 (19)
N2—C6—C7—C8	4.0 (3)	C17—C18—C19—C20	−177.4 (2)
C2—C3—C4—C5	0.0 (3)	C18—N4—C22—C21	−0.7 (3)
N3—C13—C14—C15	0.7 (3)	C18—N4—C22—C24	179.95 (19)
N3—C17—C18—N4	−6.1 (3)	C18—C19—C20—C21	−0.2 (3)
N3—C17—C18—C19	172.72 (19)	C19—C20—C21—C22	−1.4 (4)
C3—C4—C5—N1	−1.7 (3)	C20—C21—C22—N4	1.8 (3)
C3—C4—C5—C6	176.86 (19)	C20—C21—C22—C24	−178.8 (2)
N4—C18—C19—C20	1.3 (3)	C22—N4—C18—C17	177.93 (18)
C4—C5—C6—N2	178.58 (18)	C22—N4—C18—C19	−0.9 (3)
C4—C5—C6—C7	−3.4 (3)	C23—C13—C14—C15	−179.0 (2)

Symmetry code: (i) −*x*, −*y*+2, −*z*+2.

### Bis[bis(6,6'-dimethyl-2,2'-bipyridine)copper(I)] hexafluoridozirconate(IV) 1.134-hydrate (I) Hydrogen-bond geometry (Å, º)

**Table:**

*D*—H···*A*	*D*—H	H···*A*	*D*···*A*	*D*—H···*A*
O1—H1*B*···F2	0.87	1.47	2.337 (4)	177

### Bis[bis(6,6'-dimethyl-2,2'-bipyridine)copper(I)] hexafluoridohafnate(IV) 0.671-hydrate (II) Crystal data

**Table:**

[Cu(C_12_H_12_N_2_)_2_]_2_[HfF_6_]·0.671H_2_O	*Z* = 1
*M**_r_* = 1168.58	*F*(000) = 583
Triclinic, *P*1	*D*_x_ = 1.745 Mg m^−^^3^
*a* = 8.5737 (1) Å	Mo *K*α radiation, λ = 0.71073 Å
*b* = 10.7967 (2) Å	Cell parameters from 31642 reflections
*c* = 13.0183 (2) Å	θ = 2.2–33.9°
α = 103.273 (1)°	µ = 3.35 mm^−^^1^
β = 103.662 (1)°	*T* = 100 K
γ = 98.785 (1)°	Plate, clear orangish red
*V* = 1112.07 (3) Å^3^	0.3 × 0.17 × 0.08 mm

### Bis[bis(6,6'-dimethyl-2,2'-bipyridine)copper(I)] hexafluoridohafnate(IV) 0.671-hydrate (II) Data collection

**Table:**

Rigaku Saturn724+ (2x2 bin mode) diffractometer	8003 independent reflections
Radiation source: Rotating Anode, Rotating Anode	7235 reflections with *I* > 2σ(*I*)
Confocal monochromator	*R*_int_ = 0.032
Detector resolution: 28.5714 pixels mm^-1^	θ_max_ = 33.9°, θ_min_ = 2.0°
profile data from ω–scans	*h* = −13→13
Absorption correction: gaussian (CrysAlisPro; Rigaku OD, 2020)	*k* = −16→16
*T*_min_ = 0.433, *T*_max_ = 1.000	*l* = −19→19
40796 measured reflections	

### Bis[bis(6,6'-dimethyl-2,2'-bipyridine)copper(I)] hexafluoridohafnate(IV) 0.671-hydrate (II) Refinement

**Table:**

Refinement on *F*^2^	Primary atom site location: dual
Least-squares matrix: full	Hydrogen site location: mixed
*R*[*F*^2^ > 2σ(*F*^2^)] = 0.023	H-atom parameters constrained
*wR*(*F*^2^) = 0.056	*w* = 1/[σ^2^(*F*_o_^2^) + (0.029*P*)^2^ + 0.1688*P*] where *P* = (*F*_o_^2^ + 2*F*_c_^2^)/3
*S* = 1.08	(Δ/σ)_max_ = 0.002
8003 reflections	Δρ_max_ = 0.48 e Å^−^^3^
312 parameters	Δρ_min_ = −0.73 e Å^−^^3^
0 restraints	

### Bis[bis(6,6'-dimethyl-2,2'-bipyridine)copper(I)] hexafluoridohafnate(IV) 0.671-hydrate (II) Special details

**Table:**

Geometry. All esds (except the esd in the dihedral angle between two l.s. planes) are estimated using the full covariance matrix. The cell esds are taken into account individually in the estimation of esds in distances, angles and torsion angles; correlations between esds in cell parameters are only used when they are defined by crystal symmetry. An approximate (isotropic) treatment of cell esds is used for estimating esds involving l.s. planes.

### Bis[bis(6,6'-dimethyl-2,2'-bipyridine)copper(I)] hexafluoridohafnate(IV) 0.671-hydrate (II) Fractional atomic coordinates and isotropic or equivalent isotropic displacement parameters (Å^2^)

**Table:**

	*x*	*y*	*z*	*U*_iso_*/*U*_eq_	Occ. (<1)
Cu1	0.30255 (2)	0.35382 (2)	0.69812 (2)	0.01849 (4)	
N1	0.49323 (15)	0.36487 (11)	0.82812 (9)	0.0157 (2)	
C1	0.55897 (18)	0.26589 (14)	0.85279 (11)	0.0184 (3)	
N2	0.33991 (15)	0.54691 (12)	0.77752 (10)	0.0187 (2)	
C2	0.70930 (18)	0.28901 (15)	0.93195 (12)	0.0200 (3)	
H2	0.752847	0.218202	0.949428	0.024*	
N3	0.08393 (15)	0.23090 (11)	0.61730 (10)	0.0173 (2)	
C3	0.79431 (18)	0.41592 (16)	0.98475 (12)	0.0218 (3)	
H3	0.898138	0.433093	1.037654	0.026*	
N4	0.33880 (17)	0.29739 (13)	0.54432 (11)	0.0211 (2)	
C4	0.72716 (18)	0.51810 (15)	0.96003 (12)	0.0206 (3)	
H4	0.784176	0.605989	0.995319	0.025*	
C5	0.57452 (16)	0.48929 (13)	0.88246 (11)	0.0157 (2)	
C6	0.48691 (17)	0.59126 (13)	0.85563 (11)	0.0159 (2)	
C7	0.54737 (18)	0.72313 (14)	0.90906 (12)	0.0196 (3)	
H7	0.653940	0.753278	0.959803	0.024*	
C8	0.4488 (2)	0.81009 (14)	0.88679 (13)	0.0222 (3)	
H8	0.487246	0.900700	0.922570	0.027*	
C9	0.2948 (2)	0.76402 (15)	0.81239 (13)	0.0240 (3)	
H9	0.223979	0.821769	0.799097	0.029*	
C10	0.24454 (19)	0.63174 (15)	0.75708 (14)	0.0247 (3)	
C11	0.4606 (2)	0.13042 (15)	0.79400 (14)	0.0287 (3)	
H11A	0.374850	0.108709	0.829125	0.043*	
H11B	0.533164	0.068661	0.797454	0.043*	
H11C	0.409525	0.125179	0.716942	0.043*	
C12	0.0821 (3)	0.5765 (2)	0.6712 (2)	0.0523 (7)	
H12A	0.100542	0.546189	0.599023	0.078*	
H12B	0.017881	0.644134	0.669871	0.078*	
H12C	0.021671	0.503124	0.688661	0.078*	
C13	−0.03848 (19)	0.20159 (14)	0.66175 (13)	0.0217 (3)	
C14	−0.1938 (2)	0.13117 (16)	0.59615 (16)	0.0294 (3)	
H14	−0.279577	0.111864	0.628577	0.035*	
C15	−0.2222 (2)	0.08956 (17)	0.48344 (16)	0.0331 (4)	
H15	−0.328350	0.042958	0.437675	0.040*	
C16	−0.0952 (2)	0.11615 (16)	0.43760 (14)	0.0279 (3)	
H16	−0.112119	0.086313	0.360420	0.034*	
C17	0.05785 (19)	0.18743 (14)	0.50662 (12)	0.0196 (3)	
C18	0.2030 (2)	0.21916 (14)	0.46699 (12)	0.0215 (3)	
C19	0.2026 (3)	0.16910 (16)	0.35783 (13)	0.0293 (3)	
H19	0.105673	0.115925	0.304363	0.035*	
C20	0.3465 (3)	0.19880 (19)	0.32964 (15)	0.0370 (4)	
H20	0.349867	0.165652	0.256178	0.044*	
C21	0.4854 (3)	0.27688 (19)	0.40872 (16)	0.0348 (4)	
H21	0.585269	0.296727	0.390165	0.042*	
C22	0.4786 (2)	0.32667 (16)	0.51629 (14)	0.0265 (3)	
C23	0.0002 (2)	0.24806 (18)	0.78440 (14)	0.0296 (3)	
H23A	0.107943	0.232844	0.818103	0.044*	
H23B	−0.084204	0.200229	0.808740	0.044*	
H23C	0.002281	0.341524	0.806634	0.044*	
C24	0.6249 (2)	0.41315 (19)	0.60455 (16)	0.0332 (4)	
H24A	0.593380	0.490705	0.642476	0.050*	
H24B	0.711624	0.439691	0.571619	0.050*	
H24C	0.665684	0.365753	0.657414	0.050*	
Hf1	0.000000	1.000000	1.000000	0.02373 (3)	
F1	0.09274 (14)	0.99551 (11)	0.87132 (10)	0.0369 (2)	
F2	−0.05307 (12)	0.80472 (9)	0.95921 (9)	0.0312 (2)	
F3	0.22038 (12)	1.00043 (10)	1.09439 (10)	0.0341 (2)	
O1	−0.1061 (5)	0.6264 (4)	0.8063 (3)	0.0338 (12)	0.336 (5)
H1A	−0.014695	0.600372	0.823726	0.051*	0.336 (5)
H1B	−0.097233	0.702606	0.850509	0.051*	0.336 (5)

### Bis[bis(6,6'-dimethyl-2,2'-bipyridine)copper(I)] hexafluoridohafnate(IV) 0.671-hydrate (II) Atomic displacement parameters (Å^2^)

**Table:**

	*U*^11^	*U*^22^	*U*^33^	*U*^12^	*U*^13^	*U*^23^
Cu1	0.01904 (8)	0.01683 (8)	0.01581 (8)	0.00282 (6)	−0.00052 (6)	0.00350 (6)
N1	0.0167 (5)	0.0146 (5)	0.0154 (5)	0.0047 (4)	0.0031 (4)	0.0040 (4)
C1	0.0211 (6)	0.0187 (6)	0.0178 (6)	0.0077 (5)	0.0063 (5)	0.0067 (5)
N2	0.0177 (5)	0.0158 (5)	0.0213 (6)	0.0045 (4)	0.0018 (4)	0.0058 (4)
C2	0.0208 (6)	0.0249 (7)	0.0215 (7)	0.0115 (5)	0.0098 (5)	0.0119 (5)
N3	0.0197 (5)	0.0142 (5)	0.0161 (5)	0.0046 (4)	0.0013 (4)	0.0035 (4)
C3	0.0148 (6)	0.0293 (7)	0.0227 (7)	0.0063 (5)	0.0039 (5)	0.0105 (6)
N4	0.0247 (6)	0.0214 (6)	0.0215 (6)	0.0094 (5)	0.0074 (5)	0.0106 (5)
C4	0.0159 (6)	0.0207 (7)	0.0214 (7)	0.0025 (5)	0.0015 (5)	0.0032 (5)
C5	0.0144 (6)	0.0165 (6)	0.0169 (6)	0.0044 (4)	0.0051 (5)	0.0044 (5)
C6	0.0159 (6)	0.0154 (6)	0.0180 (6)	0.0043 (4)	0.0063 (5)	0.0054 (5)
C7	0.0200 (6)	0.0163 (6)	0.0220 (7)	0.0022 (5)	0.0071 (5)	0.0041 (5)
C8	0.0320 (8)	0.0141 (6)	0.0246 (7)	0.0061 (5)	0.0142 (6)	0.0061 (5)
C9	0.0280 (7)	0.0199 (7)	0.0328 (8)	0.0125 (6)	0.0136 (6)	0.0145 (6)
C10	0.0219 (7)	0.0203 (7)	0.0332 (8)	0.0067 (5)	0.0037 (6)	0.0125 (6)
C11	0.0364 (9)	0.0170 (7)	0.0280 (8)	0.0082 (6)	−0.0004 (7)	0.0053 (6)
C12	0.0307 (10)	0.0317 (10)	0.0783 (16)	0.0056 (8)	−0.0207 (10)	0.0220 (10)
C13	0.0216 (7)	0.0166 (6)	0.0263 (7)	0.0054 (5)	0.0059 (6)	0.0049 (5)
C14	0.0206 (7)	0.0206 (7)	0.0439 (10)	0.0028 (6)	0.0077 (7)	0.0053 (7)
C15	0.0228 (7)	0.0220 (8)	0.0402 (10)	0.0018 (6)	−0.0064 (7)	−0.0012 (7)
C16	0.0306 (8)	0.0215 (7)	0.0219 (7)	0.0053 (6)	−0.0062 (6)	0.0012 (6)
C17	0.0249 (7)	0.0149 (6)	0.0167 (6)	0.0070 (5)	0.0000 (5)	0.0041 (5)
C18	0.0308 (8)	0.0182 (6)	0.0162 (6)	0.0092 (6)	0.0036 (6)	0.0071 (5)
C19	0.0483 (10)	0.0248 (8)	0.0169 (7)	0.0136 (7)	0.0084 (7)	0.0073 (6)
C20	0.0652 (13)	0.0345 (9)	0.0250 (8)	0.0232 (9)	0.0248 (9)	0.0139 (7)
C21	0.0474 (11)	0.0339 (9)	0.0380 (10)	0.0185 (8)	0.0264 (9)	0.0177 (8)
C22	0.0308 (8)	0.0270 (8)	0.0318 (8)	0.0131 (6)	0.0155 (7)	0.0162 (6)
C23	0.0297 (8)	0.0349 (9)	0.0272 (8)	0.0077 (7)	0.0141 (7)	0.0077 (7)
C24	0.0258 (8)	0.0366 (9)	0.0427 (10)	0.0071 (7)	0.0154 (7)	0.0154 (8)
Hf1	0.01372 (4)	0.02043 (5)	0.04096 (6)	0.00568 (3)	0.00576 (3)	0.01683 (4)
F1	0.0372 (6)	0.0331 (6)	0.0534 (7)	0.0157 (5)	0.0229 (5)	0.0215 (5)
F2	0.0218 (5)	0.0179 (4)	0.0518 (6)	0.0039 (3)	0.0038 (4)	0.0125 (4)
F3	0.0193 (4)	0.0218 (5)	0.0541 (7)	0.0064 (4)	−0.0039 (4)	0.0100 (4)
O1	0.036 (2)	0.035 (2)	0.0227 (19)	0.0034 (16)	0.0075 (15)	−0.0029 (15)

### Bis[bis(6,6'-dimethyl-2,2'-bipyridine)copper(I)] hexafluoridohafnate(IV) 0.671-hydrate (II) Geometric parameters (Å, º)

**Table:**

Cu1—N1	2.0229 (12)	C12—H12B	0.9800
Cu1—N2	2.0414 (12)	C12—H12C	0.9800
Cu1—N3	2.0121 (12)	C13—C14	1.391 (2)
Cu1—N4	2.0659 (13)	C13—C23	1.497 (2)
N1—C1	1.3487 (17)	C14—H14	0.9500
N1—C5	1.3548 (18)	C14—C15	1.382 (3)
C1—C2	1.393 (2)	C15—H15	0.9500
C1—C11	1.497 (2)	C15—C16	1.384 (3)
N2—C6	1.3558 (18)	C16—H16	0.9500
N2—C10	1.3476 (19)	C16—C17	1.392 (2)
C2—H2	0.9500	C17—C18	1.479 (2)
C2—C3	1.381 (2)	C18—C19	1.399 (2)
N3—C13	1.344 (2)	C19—H19	0.9500
N3—C17	1.3607 (19)	C19—C20	1.381 (3)
C3—H3	0.9500	C20—H20	0.9500
C3—C4	1.386 (2)	C20—C21	1.380 (3)
N4—C18	1.356 (2)	C21—H21	0.9500
N4—C22	1.347 (2)	C21—C22	1.399 (2)
C4—H4	0.9500	C22—C24	1.495 (3)
C4—C5	1.3915 (19)	C23—H23A	0.9800
C5—C6	1.4845 (19)	C23—H23B	0.9800
C6—C7	1.389 (2)	C23—H23C	0.9800
C7—H7	0.9500	C24—H24A	0.9800
C7—C8	1.390 (2)	C24—H24B	0.9800
C8—H8	0.9500	C24—H24C	0.9800
C8—C9	1.380 (2)	Hf1—F1^i^	2.0111 (11)
C9—H9	0.9500	Hf1—F1	2.0111 (11)
C9—C10	1.392 (2)	Hf1—F2^i^	2.0033 (9)
C10—C12	1.500 (2)	Hf1—F2	2.0033 (9)
C11—H11A	0.9800	Hf1—F3^i^	1.9945 (10)
C11—H11B	0.9800	Hf1—F3	1.9945 (10)
C11—H11C	0.9800	O1—H1A	0.8700
C12—H12A	0.9800	O1—H1B	0.8699
			
N1—Cu1—N2	81.22 (5)	H12B—C12—H12C	109.5
N1—Cu1—N4	116.52 (5)	N3—C13—C14	121.15 (15)
N2—Cu1—N4	120.35 (5)	N3—C13—C23	116.98 (14)
N3—Cu1—N1	136.20 (5)	C14—C13—C23	121.87 (15)
N3—Cu1—N2	126.39 (5)	C13—C14—H14	120.3
N3—Cu1—N4	80.94 (5)	C15—C14—C13	119.42 (17)
C1—N1—Cu1	127.24 (10)	C15—C14—H14	120.3
C1—N1—C5	119.15 (12)	C14—C15—H15	120.2
C5—N1—Cu1	112.66 (9)	C14—C15—C16	119.63 (15)
N1—C1—C2	121.40 (13)	C16—C15—H15	120.2
N1—C1—C11	116.99 (13)	C15—C16—H16	120.6
C2—C1—C11	121.58 (13)	C15—C16—C17	118.80 (15)
C6—N2—Cu1	112.15 (9)	C17—C16—H16	120.6
C10—N2—Cu1	128.64 (10)	N3—C17—C16	121.28 (15)
C10—N2—C6	119.08 (13)	N3—C17—C18	115.28 (13)
C1—C2—H2	120.3	C16—C17—C18	123.44 (14)
C3—C2—C1	119.31 (13)	N4—C18—C17	115.53 (13)
C3—C2—H2	120.3	N4—C18—C19	121.93 (16)
C13—N3—Cu1	125.50 (10)	C19—C18—C17	122.51 (15)
C13—N3—C17	119.68 (13)	C18—C19—H19	120.8
C17—N3—Cu1	114.43 (10)	C20—C19—C18	118.41 (17)
C2—C3—H3	120.2	C20—C19—H19	120.8
C2—C3—C4	119.57 (13)	C19—C20—H20	120.2
C4—C3—H3	120.2	C21—C20—C19	119.67 (16)
C18—N4—Cu1	113.01 (10)	C21—C20—H20	120.2
C22—N4—Cu1	127.54 (11)	C20—C21—H21	120.1
C22—N4—C18	119.43 (14)	C20—C21—C22	119.72 (18)
C3—C4—H4	120.7	C22—C21—H21	120.1
C3—C4—C5	118.63 (13)	N4—C22—C21	120.82 (17)
C5—C4—H4	120.7	N4—C22—C24	117.38 (15)
N1—C5—C4	121.89 (13)	C21—C22—C24	121.80 (16)
N1—C5—C6	115.22 (12)	C13—C23—H23A	109.5
C4—C5—C6	122.86 (13)	C13—C23—H23B	109.5
N2—C6—C5	115.48 (12)	C13—C23—H23C	109.5
N2—C6—C7	121.72 (13)	H23A—C23—H23B	109.5
C7—C6—C5	122.77 (13)	H23A—C23—H23C	109.5
C6—C7—H7	120.7	H23B—C23—H23C	109.5
C6—C7—C8	118.65 (14)	C22—C24—H24A	109.5
C8—C7—H7	120.7	C22—C24—H24B	109.5
C7—C8—H8	120.2	C22—C24—H24C	109.5
C9—C8—C7	119.60 (14)	H24A—C24—H24B	109.5
C9—C8—H8	120.2	H24A—C24—H24C	109.5
C8—C9—H9	120.5	H24B—C24—H24C	109.5
C8—C9—C10	119.06 (14)	F1^i^—Hf1—F1	180.00 (7)
C10—C9—H9	120.5	F2^i^—Hf1—F1	89.79 (5)
N2—C10—C9	121.66 (14)	F2—Hf1—F1	90.21 (5)
N2—C10—C12	116.62 (15)	F2—Hf1—F1^i^	89.79 (5)
C9—C10—C12	121.71 (15)	F2^i^—Hf1—F1^i^	90.21 (5)
C1—C11—H11A	109.5	F2—Hf1—F2^i^	180.0
C1—C11—H11B	109.5	F3—Hf1—F1	90.15 (5)
C1—C11—H11C	109.5	F3—Hf1—F1^i^	89.85 (5)
H11A—C11—H11B	109.5	F3^i^—Hf1—F1^i^	90.15 (5)
H11A—C11—H11C	109.5	F3^i^—Hf1—F1	89.85 (5)
H11B—C11—H11C	109.5	F3—Hf1—F2	89.27 (4)
C10—C12—H12A	109.5	F3^i^—Hf1—F2^i^	89.26 (4)
C10—C12—H12B	109.5	F3^i^—Hf1—F2	90.74 (4)
C10—C12—H12C	109.5	F3—Hf1—F2^i^	90.73 (4)
H12A—C12—H12B	109.5	F3—Hf1—F3^i^	180.0
H12A—C12—H12C	109.5	H1A—O1—H1B	109.5
			
Cu1—N1—C1—C2	167.25 (11)	C5—N1—C1—C2	−0.7 (2)
Cu1—N1—C1—C11	−14.69 (19)	C5—N1—C1—C11	177.33 (13)
Cu1—N1—C5—C4	−167.08 (11)	C5—C6—C7—C8	−173.20 (13)
Cu1—N1—C5—C6	14.67 (15)	C6—N2—C10—C9	1.4 (2)
Cu1—N2—C6—C5	−11.14 (15)	C6—N2—C10—C12	−178.95 (17)
Cu1—N2—C6—C7	171.02 (11)	C6—C7—C8—C9	−0.3 (2)
Cu1—N2—C10—C9	−173.90 (12)	C7—C8—C9—C10	−3.1 (2)
Cu1—N2—C10—C12	5.7 (2)	C8—C9—C10—N2	2.6 (2)
Cu1—N3—C13—C14	170.17 (12)	C8—C9—C10—C12	−176.97 (18)
Cu1—N3—C13—C23	−9.84 (19)	C10—N2—C6—C5	172.79 (13)
Cu1—N3—C17—C16	−171.27 (11)	C10—N2—C6—C7	−5.1 (2)
Cu1—N3—C17—C18	9.88 (15)	C11—C1—C2—C3	−179.25 (14)
Cu1—N4—C18—C17	−1.01 (15)	C13—N3—C17—C16	2.0 (2)
Cu1—N4—C18—C19	−179.23 (12)	C13—N3—C17—C18	−176.87 (12)
Cu1—N4—C22—C21	177.63 (12)	C13—C14—C15—C16	1.3 (3)
Cu1—N4—C22—C24	−2.0 (2)	C14—C15—C16—C17	−1.6 (2)
N1—C1—C2—C3	−1.3 (2)	C15—C16—C17—N3	0.0 (2)
N1—C5—C6—N2	−2.27 (18)	C15—C16—C17—C18	178.70 (15)
N1—C5—C6—C7	175.55 (13)	C16—C17—C18—N4	175.36 (14)
C1—N1—C5—C4	2.6 (2)	C16—C17—C18—C19	−6.4 (2)
C1—N1—C5—C6	−175.69 (12)	C17—N3—C13—C14	−2.3 (2)
C1—C2—C3—C4	1.5 (2)	C17—N3—C13—C23	177.72 (13)
N2—C6—C7—C8	4.5 (2)	C17—C18—C19—C20	−176.75 (14)
C2—C3—C4—C5	0.3 (2)	C18—N4—C22—C21	−0.3 (2)
N3—C13—C14—C15	0.6 (2)	C18—N4—C22—C24	−179.92 (14)
N3—C17—C18—N4	−5.82 (18)	C18—C19—C20—C21	−0.4 (3)
N3—C17—C18—C19	172.39 (13)	C19—C20—C21—C22	−0.9 (3)
C3—C4—C5—N1	−2.3 (2)	C20—C21—C22—N4	1.3 (3)
C3—C4—C5—C6	175.77 (13)	C20—C21—C22—C24	−179.15 (16)
N4—C18—C19—C20	1.4 (2)	C22—N4—C18—C17	177.22 (13)
C4—C5—C6—N2	179.49 (13)	C22—N4—C18—C19	−1.0 (2)
C4—C5—C6—C7	−2.7 (2)	C23—C13—C14—C15	−179.34 (16)

Symmetry code: (i) −*x*, −*y*+2, −*z*+2.

### Bis[bis(6,6'-dimethyl-2,2'-bipyridine)copper(I)] hexafluoridohafnate(IV) 0.671-hydrate (II) Hydrogen-bond geometry (Å, º)

**Table:**

*D*—H···*A*	*D*—H	H···*A*	*D*···*A*	*D*—H···*A*
O1B—H1*B*···F2	0.87	1.50	2.328 (4)	156

### Coordination geometry (Å, °) of Cu(dmbpy)^2+^ cations in (I)

**Table:**

N—Cu—N	N—Cu	Cu—N	N—Cu—N
N1—Cu1—N2	2.0208 (16)	2.0348 (17)	81.40 (7)
N1—Cu1—N3		2.0123 (17)	136.29 (7)
N1—Cu1—N4		2.0616 (18)	116.24 (7)
N2—Cu1—N3			126.17 (7)
N2—Cu1—N4			120.45 (7)
N3—Cu1—N4			81.05 (7)

### Bond distances (Å) of ZrF_6_^2–^ in (I)

**Table:**

Zr—F	Distance (Å)
Zr—F1	2.0113 (15)
Zr—F2	2.0183 (12)
Zr—F3	1.9955 (13)

### Hydrogen-bond geometry (Å, °) for (I)

**Table:**

D—H···A	D—H	H···A	D···A	D—H···A
O1—H1B···F2	0.870 (3)	1.4674 (14)	2.337 (3)	177.1 (2)

### Coordination geometry (Å, °) of Cu(dmbpy)^2+^ cations in (II)

**Table:**

N—Cu—N	N—Cu	Cu—N	N—Cu—N
N1—Cu1—N2	2.0229 (12)	2.0414 (12)	81.22 (5)
N1—Cu1—N3		2.0121 (12)	136.20 (5)
N1—Cu1—N4		2.0659 (13)	116.52 (5)
N2—Cu1—N3			126.39 (5)
N2—Cu1—N4			120.35 (5)
N3—Cu1—N4			80.94 (5)

### Bond distances (Å) of HfF_6_^2–^ in (II)

**Table:**

Hf—F	Distance (Å)
Hf—F1	2.0111 (11)
Hf—F2	2.0033 (9)
Hf—F3	1.9945 (10)

### Hydrogen-bond geometry (Å, °) for (II)

**Table:**

D—H···A	D—H	H···A	D···A	D—H···A
O1—H1B···F2	0.870 (4)	1.5048 (11)	2.328 (4)	156.4 (3)
